# The role of PIM1/PIM2 kinases in tumors of the male reproductive system

**DOI:** 10.1038/srep38079

**Published:** 2016-11-30

**Authors:** Manuel Pedro Jiménez-García, Antonio Lucena-Cacace, María José Robles-Frías, Maja Narlik-Grassow, Carmen Blanco-Aparicio, Amancio Carnero

**Affiliations:** 1 Instituto de Biomedicina de Sevilla, IBIS/Hospital Universitario Virgen del Rocío/Universidad de Sevilla/Consejo Superior de Investigaciones Científicas, Avda. Manuel Siurot s/n 41013, Seville, Spain; 2 Experimental Therapeutics Programme, Spanish National Cancer Centre (CNIO), C/Melchor Fernández Almagro 3, 28029, Madrid, Spain

## Abstract

The PIM family of serine/threonine kinases has three highly conserved isoforms (PIM1, PIM2 and PIM3). PIM proteins are regulated through transcription and stability by JAK/STAT pathways and are overexpressed in hematological malignancies and solid tumors. The PIM kinases possess weak oncogenic abilities, but enhance other genes or chemical carcinogens to induce tumors. We generated conditional transgenic mice that overexpress PIM1 or PIM2 in male reproductive organs and analyzed their contribution to tumorigenesis. We found an increase in alterations of sexual organs and hyperplasia in the transgenic mice correlating with inflammation. We also found that PIM1/2 are overexpressed in a subset of human male germ cells and prostate tumors correlating with inflammatory features and stem cell markers. Our data suggest that PIM1/2 kinase overexpression is a common feature of male reproductive organs tumors, which provoke tissue alterations and a large inflammatory response that may act synergistically during the process of tumorigenesis. There is also a correlation with markers of cancer stem cells, which may contribute to the therapy resistance found in tumors overexpressing PIM kinases.

The Proviral Insertion site in Moloney murine leukemia virus proteins (PIM) are a highly evolutionarily conserved family of serine/threonine kinases composed of three different isoforms (PIM1, PIM2 and PIM3). These proteins are regulated primarily through transcription and stability by pathways that are controlled by JAK/STAT transcription factors^1^,^2^, cytokines, and growth factors involved in hematopoiesis, such as interleukins, GM-CSF and G-CSF^3^,^4^. In addition, nuclear factor-κB, Jak-signal transducer and activator of transcription (STAT), ETS-related gene and hypoxia-inducible factor-1α are the primary pathways that induce PIM1 upregulation^5^,^6^,^7^,^8^. Furthermore, the stability and function of PIM kinases depend on their interaction with heat shock protein (Hsp) 90^9^,^10^, a chaperone involved in the folding and stabilization of different molecules. Hsp90 has been shown to not only to protect from ubiquitin-dependent proteasomal degradation but has also been shown to maintain the proper conformation of PIM proteins^10^. The downstream targets of PIM1 signaling are typically regulated by direct phosphorylation by PIM1. Thus far, approximately 30 substrates have been shown to interact with and be phosphorylated by PIM1. Through phosphorylation of target proteins, PIM1 plays essential roles in the regulation of the cell cycle, cell proliferation, anti-apoptosis, multiple drug resistance, chromatin remodeling, protein translation, energy metabolism, and the stress response^11^,^12^,^13^. It has been suggested that PIM family members are weak oncogenes that can contribute to tumorigenesis by selectively enhancing tumorigenic properties^1^,^2^. However, PIM family members have also been shown to increase the capacity of other genes or chemical carcinogens to induce tumors^14^,^15^,^16^. The magnitude of the effect varies depending on the affected tissues and the pathways that are activated.

Different members of the PIM family of kinases have been found to be overexpressed in hematological malignancies and solid tumors (reviewed at refs 1, 2, 3, 4), which led to the idea that these proteins could be potentially interesting targets for anticancer drug therapy^17^,^18^,^19^,^20^. PIM1 has been observed to be overexpressed in numerous solid tumors^5^,^21^,^22^,^23^, including prostate^24^ and testicular tumors^2^. Recent studies have correlated PIM1 kinase with chemoresistance in prostate cancer cells, which is a common feature of the more aggressive, hormone-refractory prostate cancers^25^,^26^, and accordingly, the expression of PIM1 correlated with a poor therapeutic outcome^27^. It has been reported that PIM1 kinase, facilitates cell survival in hypoxia-induced genetic instability in solid tumors and resulting in a more aggressive phenotype^28^. Increased PIM1 expression could partly explain the strong resistance of these cancers to chemotherapy^29^.

Increased PIM2 kinase levels have been detected in acute myeloid leukemia patients and B-cell derived malignancies^30^,^31^. Furthermore, PIM2 increased levels have also been shown to be associated with aggressive clinical outcome in ABC-DLBCL patients^32^,^33^. PIM2 levels have also been found to be elevated in prostate cancers and have been correlated with high proliferation rates and decreased apoptosis^34^.

Aberrant expression of the PIM3 kinase has been observed in malignant tumors of the liver and the pancreas as well as. PIM3 has been shown to be highly expressed in Ewing’s sarcoma, hepatocellular carcinoma and pancreatic cancer^11^,^35^,^36^,^37^. Finally, in some cancers, such as germ cell derived tumors, all three PIM members have been found to be overexpressed^2^. Furthermore, in these cancer types, PIM1 and PIM2 can be overexpressed in the same tumors, which suggest that there is only a partial redundancy among them and share some common physiological properties.

To characterize the proto-oncogenic role of PIM1/PIM2 in the male reproductive system, we generated two MMTV-Cre conditional transgenic mice with confined expression of human PIM1 or PIM2 in hormone-dependent tissues. We fully characterized the tumor responses to these genetic alterations in both PIM1 and PIM2 models, corroborating their role as oncogenes by inducing a hyperproliferation state in tissues of the male reproductive system.

## Materials and Methods

All methods were performed in accordance with the relevant guidelines and regulations of the Institute for Biomedical Research of Seville (IBIS) and University Hospital Virgen del Rocio (HUVR).

### Maintenance of mouse colonies

All of the experiments performed using animals received the expressed approval from the IBIS/HUVR Ethical Committee for the Care and Health of Animals. All of the animals were kept in the IBIS animal facility according to the facility guidelines, which are based on the Real Decreto 53/2013 and were sacrificed by CO_2_ inhalation either within a programmed procedure or as a humane endpoint when the animals showed significant signs of illness. Efforts were made to minimize suffering wherever possible.

### Construction of the transgenic DNA

**Figure 1:**
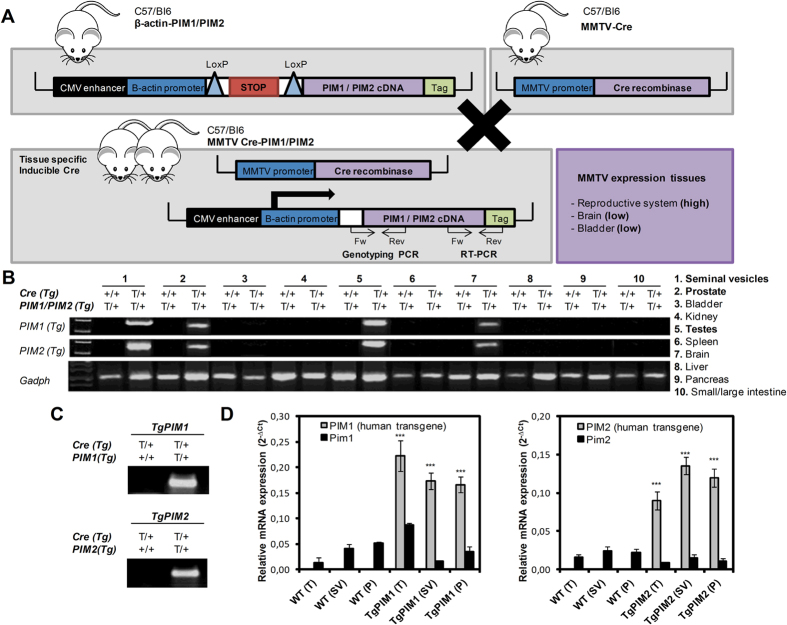
The generation and validation of the conditional transgenic MMTV-Cre/PIM1 and MMTV-Cre/PIM2 murine models. (**A**) The cDNA sequences of human PIM1 and PIM2 were amplified by PCR using specifically designed primers of cDNA from human IMR90 cells as a template. Human PIM1 and PIM2 genes were then cloned into the pVL-1 vector, which was a gift from A. Nebreda. The animals carrying the respective transgenes for PIM1 or PIM2 were crossed with mice expressing Cre-recombinase under the regulation of the MMTV promoter, which resulted in two mouse lines that expressed either PIM1 or PIM2 human transgenes exclusively in hormone-dependent tissues. (**B**) Reverse transcription PCR was performed to validate the tissue-specific, relative expression levels of either PIM1 or PIM2 in the transgenic models to confirm transgene expression in tissue from the seminal vesicle, prostate, testicle, and brain. (**C**) DNA obtained from the digested tails of the mice was used to genotype the TgPIM1 or TgPIM2 models by PCR. (**D**) The levels of the human PIM1 and PIM2 transgenes and mouse endogenous Pim1 and Pim2 mRNAs were measured by qRT-PCR in seminal vesicle (SV), prostate (P) and testicle (T) from both TgPIM1 and TgPIM2 and was compared to WT mice. The p-value was obtained using a one-tailed student’s t-test. (*p < 0.05), (**p < 0.01), and (***p < 0.001).

Human PIM1 and PIM2 cDNAs were cloned into the pVL-1 vector (Fig. 1A). The DNA construct was linearized and injected into embryonic stem cells. The embryonic stem cell injection, selection and transfer were performed by the Transgenic Mice Unit of the CNIO according to their standard protocols, as previously described^38^. Mice born after embryonic stem cell injection were genotyped using specifically designed primers, crossed with wild-type (WT) C57/Bl6 mice, and the resultant pups were genotyped to verify germ line transmission. Founder mice were then bred, and conditional transgene expression was verified by RT-PCR. Primers designed specifically for human PIM1 and PIM2 kinases, which do not amplify mouse Pim genes, were used for all PCR experiments and subsequent genotyping of mice. The primers used were, for human genes: PIM1Fw: 5′: CGAGATCGCCATATTTGGTGTCCCCGAG; Rv: 5′: CCAGCTTGGTGGCGTGCAGGTCGTTGCA; PIM2 Fw: 5′: GGCAGCCAGCATATGGG; Rv: 5′: TAATCCGCCGGTGCCTGG. For mouse genes: Pim1 Fw: 5′: CAAGGACGAAAACATCCTTATC; Rv: 5′: GATGGGACCCGAGTGTATAGCC; Pim2 Fw: 5′: GGCAGCCAGCATATGGGC; Rv: 5′: TAATCCGCCGGTGCCTGG.

### Necropsy, pathological studies, and immunohistochemistry

Mice were sacrificed by CO2 inhalation, and all tissues and tumor samples were immediately dissected and fixed in 10% buffered formalin for 24 h, dehydrated at different ethanol concentrations with xylol and embedded in paraffin at 65 °C to obtain tissue blocks. For histopathological analysis, paraffin blocks were cut into 2.5 μm sections, mounted and dried on glass slides. Sectioned tissues were deparaffinized in xylol, followed by dehydration in graded alcohol solutions and were stained with hematoxylin-eosin (H&E).

Histopathologic evaluation of the male reproductive system of mice was performed based on the criteria of InHand Project (International Harmonization of Nomenclature and Diagnostic Criteria for lesions in Rats and Mice) in relation to proliferative and non-proliferative lesions of the male reproductive system and rat mouse (Proliferative and Non Proliferative lesions of the rat and mouse male reproductive system Toxicologic Pathology. 40: 40S–121S, 2012) and based on the pathologic alterations of the prostate in genetically modified animals: classification Bar Harbor (prostate Pathology of Genetically Engineered Mice: Definitions and Classification The Consensus report from the Bar Harbor Meeting of the Mouse Models of Human Prostate Cancer Consortium Pathology Committee Cancer Research, 64: 2270–2305, 2004).

The inflammatory cell infiltration was assessed as present or absent according to the presence or absence of immune cells.

For immunostaining on sectioned tissues, epitope antigen retrieval was performed in sodium citrate (pH 6.5). Endogenous peroxidase activity was blocked using DAKO blocking solution for 20 minutes at room temperature. Non specific protein binding was saturated using PBS solution containing 10% fetal bovine serum, 1% bovine serum albumin and 0.3% Triton X-100 for 1 h at room temperature. Anti-Ki67 (DAKO) antibody was used as primary antibody, and the slides were incubated overnight at 4 °C. A secondary antibody anti-goat (ab97100) for: CD3 (sc-1127); anti-rat (JI-112-035-003) for: CD45 (NB110-93609), F4/80 (MCA497), and anti-rabbit (JI-111-035-003) for: Ki67, was applied for 1 h at room temperature, and the immunocomplexes were revealed using substrate buffer and chromogen (Envision, Flex DAKO). The tissues were counterstained with hematoxylin (DAKO), rehydrated in a graded alcohol series, and mounted using coverslips. All these procedures were carried out at the Histopathology Unit at the IBIS.

### Analysis of relative (RT-PCR) and quantitative (qRT-PCR) expression of PIM1/PIM2 mRNA transgenes and immune-dependent mRNAs

Total RNA was isolated from N2 pulverized tissues by using miRNeasy kit (Qiagen), treated with DNase (Qiagen), and reverse transcribed with random primers and reverse transcriptase (ThermoFisher), according to the manufacturer’s instructions. Reverse transcriptase PCR (RT-PCR) was performed to validate tissue specific relative expression levels of either PIM1 or PIM2 (both 500 pb band) in TgPIM1/TgPIM2 mice. The cDNA was amplified by PCR using specific primer previously mentioned. To measure mRNA expression by qRT-PCR we used the following TaqMan Gene Expression Assays probes (ThermoFisher) after RT-PCR: PIM1 (Hs01065498_m1), PIM2 (Hs00179139_m1), Pim1 (Mm00435712_m1), Pim2 (Mm00454579_m1), and endogenous housekeepings: GADPH (Hs03929097_g1) and Gadph (Mm99999915_g1). And all immune-related probes: CD4 (Mm00442754_m1), CD8a (Mm01182107_g1), CD74 (Mm00658576_m1), IFNG (Mm01168134_m1), TAP1 (Mm00443188_m1), TAP2 (Mm01277033_m1), and the main HLA in mouse: H2-DMA (Mm00439226_m1), H2-K (Mm01612247_mH), H2-A: (Mm00439211_m1), H2-E: (Mm00772352_m1). Real-time PCR was performed using an ABI 7900HT (ThermoFisher). The relative mRNA quantities were expressed as 2^−∆Ct^. Relative mRNA quantification and statistical analysis of qPCR data were conducted using RQ Manager 1.2.1 software (ThermoFisher).

### Statistical analysis

All of the grouped data are presented as the mean ± standard error. The differences between the groups were assessed by a one- or two-tailed Student’s T-test using GraphPad Prism Software. For survival analysis, Kaplan-Meier curves were generated using Prism Software, and a long-rank test was performed to determine significant differences between the groups. All of the experiments were repeated in at least duplicate with triplicate technical replicates for each condition. The data distribution was assumed to be normal, but this was not formally tested. The data obtained for retrospective analysis were collected and processed in appropriate experimental arms.

### Bioinformatic analysis

We analyzed the available, public tumor dataset for PIM kinases expression in either testicular seminoma tumors at various stages of progression (GSE8607, Chaganti dataset) or prostate tumors (GSE21034, Sawyer dataset) available at Oncomine (Compendia Biosciences, www.oncomine.org) and R2: Genomics analysis and visualization platform (http://r2.amc.nl). For analysis with high and low groups, high was defined as greater than one standard deviation above the mean, low is greater than one standard deviation below the mean. We also use an independent dataset (GSE3218, Korkola dataset) for adult male germ cell tumors. One-way ANOVA was performed to get the statistics. To study both PIM association with chemoresistance and immune factors, we used GSE8607 (Chaganti dataset), GSE21034 (Sawyer dataset), and also a tumor prostate adenocarcinoma dataset from TGCA (http://cancergenome.nih.gov/), from either PIM1 or PIM2 and the stem/immune genes activated by their expression, establishing both a stem and a immune PIM dependent signatures. Heat-maps are generated by City-block distances.

## Results

### Generation of transgenic mice carrying the PIM1 or PIM2 transgene

We generated mouse lines that express the PIM1 or PIM2 transgene specifically in the male reproductive organs by crossing our PIM1 or PIM2 transgenic lines with a transgenic line expressing Cre-recombinase under the MMTV promoter (Fig. 1A). The Lox/Stop/Lox cassette was excised by Cre-recombinase allowing transgene expression, which was tested using RT-PCR to confirm the specificity (Fig. 1B). Both transgenic lines were expressed only in the seminal vesicles, prostate, testes, and, in a small amount, in the brain (Fig. 1B). To evaluate the specificificity of the transgene we genotyped the PIM1 or PIM2 transgene. PIM transgenic cDNA was not detected in WT but was clear in PIM1 or PIM2 Transgenic mice (Fig. 1C). The levels of transgenic PIM1 or PIM2 mRNAs are around ten/twelve-fold higher on average than the levels of endogenous mouse Pim mRNAs (Fig. 1D).

### Phenotype of the transgenic mice expressing the PIM1 or PIM2 transgene

**Figure 2:**
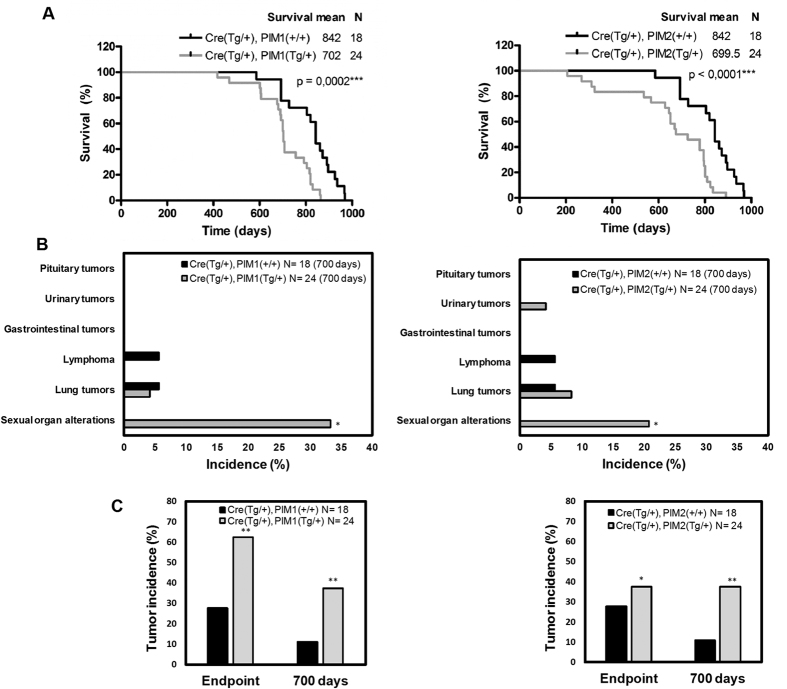
Phenotype of PIM transgenic mice. (**A**) Survival of the TgPIM1, TgPIM2, and WT mouse models. Both models show a statistically significant reduction in the survival rate compared to the WT mice. (**B**) Tumor incidence in both male models at day 700. The data show the observed macroscopic pathologies in TgPIM1 and TgPIM2 mice compared to the WT background, which indicates a statistically significant increase in tumor incidence in sex organs (testes, seminal vesicles, and prostate lobes). (**C**) Total tumor incidence in both models at day 700 and the clinical endpoint. This graph shows the total percentage of tumors observed macroscopically per model, compared to WT. The p-value was obtained using a Mantel-Cox test (also called a long-rank Chi2 based test) in (**A**), and a one-tailed student’s t-test in (**B** and **C**). (*p < 0.05), (**p < 0.01), and (***p < 0.001).

To investigate the proto-oncogenic role of human PIM1 and PIM2 proteins in male reproductive organs, we generated conditional transgenic murine cohorts of male mice that overexpressed either PIM1 or PIM2 transgenes and maintained this colony in a controlled environment. Control cohorts without transgene expression were siblings maintained under the same conditions. We observed a significant decrease in the average lifespan of the PIM transgenic cohorts (Fig. 2A). At the time of death, a macroscopic analysis showed an increase in alterations in the sex organs in the transgenic cohort compared to the control cohorts (Fig. 2B). An in-depth microscopic analysis performed at necropsy on the sex organs showed a clear increase in tumors in the transgenic mice (Fig. 2C). Specifically, up to 60% of the PIM1 and 40% of the PIM2 transgenic mice carried a tumor, while in the WT mice, the percentage of animals with tumors did not reach 30%. To reduce the frequency of hematological tumors that normally occur in elderly WT mice, we selected 700 days as the end-point. At this time point, both transgenic lines had a statistically significant increase in the percentage of total tumors compared to WT mice (Fig. 2C).

**Figure 3:**
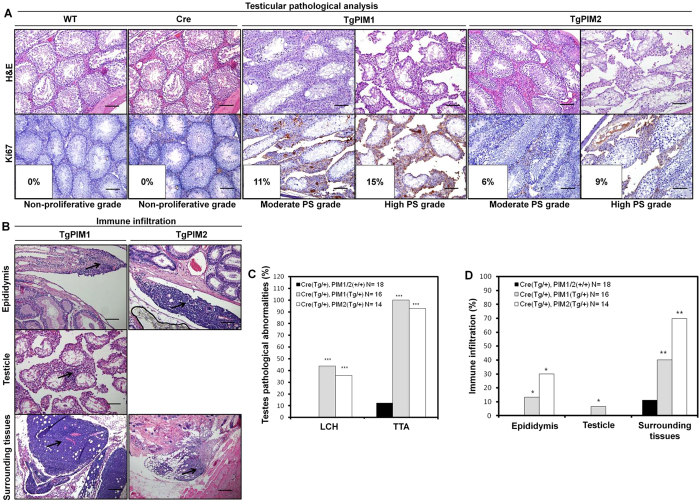
Pathological analysis of the testes from MMTV-Cre/PIM1 and MMTV-Cre/PIM2 transgenic models. (**A**) Pathological analysis of the testes was performed by H&E and Ki67 proliferation marker immunostaining of both PIM models, compared to WT. Shown are moderate and high grades of both degenerative (testicular tubular atrophy, TTA) and pre-neoplastic lesions (Leydig cells hyperplasia, LCH), which were present in only the transgenic models. Also shown is the percentage of Ki67 signal intensity. PS indicates Proliferative Status. All scale bars indicate 100 μm (20X). (**B**) Representative images of immune infiltration (marked with arrows) in germinal tissues (epididymis and testicle) from transgenic mice are shown. (**C**) Pathological abnormalities were examined, and the graph indicates the percentage of degenerative (TTA) and Leydig cells hyperplasia in the testicles of both models compared to WT mice. (**D**) The percentage of mice having immune infiltration in germinal organs and nearby tissue structures in transgenic mice was compared to WT mice. All of the images were captured using the Microscope Olympus BX-61. The p-value was obtained using a one-tailed student’s t-test. (*p < 0.05), (**p < 0.01), and (***p < 0.001).

Following the macroscopic analysis, we performed a microscopic histopathological analysis of the entire male reproductive system: testes, epididymis, seminal vesicles, and the prostatic lobes (anterior, dorsolateral, and ventral). We found different grades of both degenerative (testicular tubular atrophy, TTA) and pre-neoplastic lesions (Leydig cells hyperplasia, LCH) in all tissues from the testicles that we analyzed from the transgenic models, with a slightly higher pre-neoplastic phenotype being observed in the PIM1 mice (Fig. 3A and C).

We further analyzed the proliferation phenotype status in testicle tissues by measuring Ki67 immuno-marker levels and found a statistically significant increase in the Ki67 signal in TgPIM1/TgPIM2 mice compared to WT mice (Fig. 3A). Conversely, we did not find any relevant disturbances or alterations in tissue from the epididymis (data not shown). Interestingly, transgenic mice showed an important inflammatory component in the epididymis, testicle, and surrounding tissues (Fig. 3B and D, and Supplementary Figure S1).

**Figure 4:**
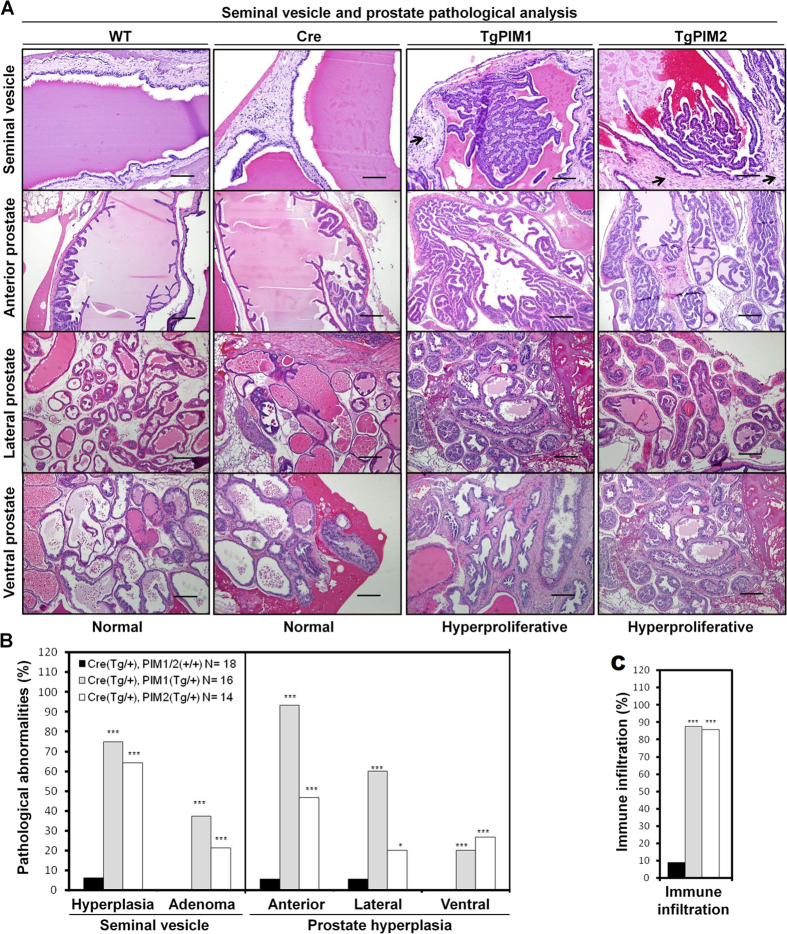
Pathological analysis of the male reproductive system in PIM1/PIM2 transgenic models. (**A**) H&E stating was used to evaluate the pathology of the seminal vesicle as well as the anterior, lateral, and ventral prostate lobes in WT and both transgenic models, which revealed normal and hyperproliferative grades, respectively. TgPIM1 and TgPIM2 seminal vesicles show areas of epithelial hyperplasia and adenomas. Arrows in TgPIM1/TgPIM2 seminal vesicles indicate immune infiltration, which was not found in WT tissues. The seminal vesicles scale bars indicates 100 μm (20X), and the anterior prostate scale bars indicate 200 μm (10X). (**B**) Following pathological examination of the seminal vesicle and prostate, a higher percentage of hyperplastic lesions were observed in both transgenic models compared to WT mice. (**C**) Immune infiltration graph indicating cell infiltration and inflammation phenotypes found in all transgenic mice and WT reproductive system samples. All of the images were captured using the Microscope Olympus BX-61. The p-value was obtained using a one-tailed student’s t-test (*p < 0.05), (**p < 0.01), and (***p < 0.001).

Additionally, we also found a pre-neoplastic phenotype in the seminal vesicles and the prostate lobes (Fig. 4). We specifically discovered hyper-proliferation and adenoma formation in the epithelial components of the seminal vesicles, which corresponded to the macroscopic results that showed darkening (brown to black) of both lobes and the contents of the seminal vesicle as well as a 2–3 time increase in the size of the seminal vesicles (Fig. 4A and B). The analysis of the prostate lobes showed that transgenic mice have a statistically significant increase in hyper-proliferation of the anterior, dorsolateral, and ventral lobes of the prostate compared to WT mice (Fig. 4A and B). Additionally, according to Shappell *et al*. 2004, classification of prostate neoplasia, they were classified as benign hyper-proliferation and also prostate intraductal neoplasia, mPIN, I focal (grade 1).

During the histopathological analysis, we observed a high degree of immune infiltration in the seminal vesicle and prostate in both transgenic cohorts but not in the WT mice (Fig. 4C and D, and Supplementary Figure S2). This relationship between the expression of the PIM kinases and the mobilization of the immune system was strong because it was observed in almost 100% of the transgenic mice.

Together our data indicate that PIM1/PIM2 over-expression induces a pre-neoplastic phenotype in the testicle, seminal vesicles, and prostate, which indicates a possible role in the initiation of oncogenesis in these tissues. Furthermore, the presence of inflammation is likely an important second signal contributing to the tumorigenesis.

### The relevance of PIM1/2 expression in human male germ cell and prostate tumors

**Figure 5:**
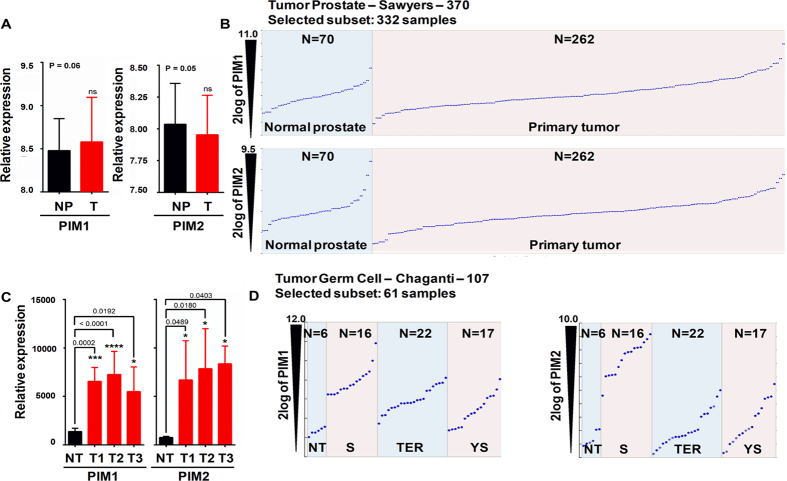
The role of PIM overexpression in male germ cell and prostate tumors. (**A**) Analysis of GSE21034 (Prostate tumor) for PIM1/2 relative expression. (Normal Prostate (NP) n = 70; Primary tumor (T) n = 262; PIM1 p = 0.06; PIM2 p = 0.05, by an unpaired t-test). (**B**) Patients were classified into groups based on the PIM1/2 gene expression level. (**C**) Analysis of available, public tumor datasets for PIM kinases expression in either testicular or seminoma tumors at various stages of progression (GSE8607) available at Oncomine and R2 platform. High and low groups were defined as above and below the mean, respectively. For analysis of the high and low groups, high was defined as greater than one standard deviation above the mean, and low was defined as greater than one standard deviation below the mean. In total, 43 samples were segregated onto Normal testis, pT1, pT2, and pT3. One-way ANOVA was performed to determine the statistical significance. PIM1: pT1, *p* = 0.0002***; pT2, *p* < 0.0001****; pT3, *p* = 0.0192*. PIM2: pT1, *p* = 0.0489*; pT2, *p* = 0.0180*; pT3, *p* = 0.0403*. (**D**) Analysis of PIM kinases expression levels in an independent dataset (GSE3218) of adult male germ cell tumors, compared to healthy tissue (NT), Seminoma (S), Teratoma (TER), and Yolk Sac (YS) tumors.

To confirm the clinical relevance of PIM kinases expression in human prostate tumors, we analyzed the available public tumor dataset for PIM kinase expression. We did not find a clear increase in the overall levels of PIM kinases in tumor samples compared to non-tumor tissue from the prostate (Fig. 5A and B). However, it is possible to detect a small subset of prostate tumors with clearly increased levels of these proteins. This subset of tumors with high PIM1/2 constitutes approximately 15% of all prostate tumors.

To confirm the clinical relevance of PIM kinases expression in human male germ cell tumors, we analyzed the available public tumor datasets (GSE8607, GSE3218) for PIM kinase expression in testicular seminoma tumors at various stages of progression available at Oncomine (Compendia Biosciences, www.oncomine.org) and R2: Genomics analysis and visualization platform (http://r2.amc.nl/). We identified that both PIM kinases are strongly overexpressed compared to healthy testis specimens and that this overexpression is independent of the tumor stage (Fig. 5C). Furthermore, PIM kinases were overexpressed in all tumor types: Seminoma, Teratoma, and Yolk Sac tumors (Fig. 5D). Interestingly, seminoma showed the highest levels of PIM1/2 kinase overexpression in the highest percentage of tumors (Fig. 5D).

### PIM1 and PIM2 expression correlates with a pro-inflammatory phenotype in human male germ cell and prostate tumors

**Figure 6:**
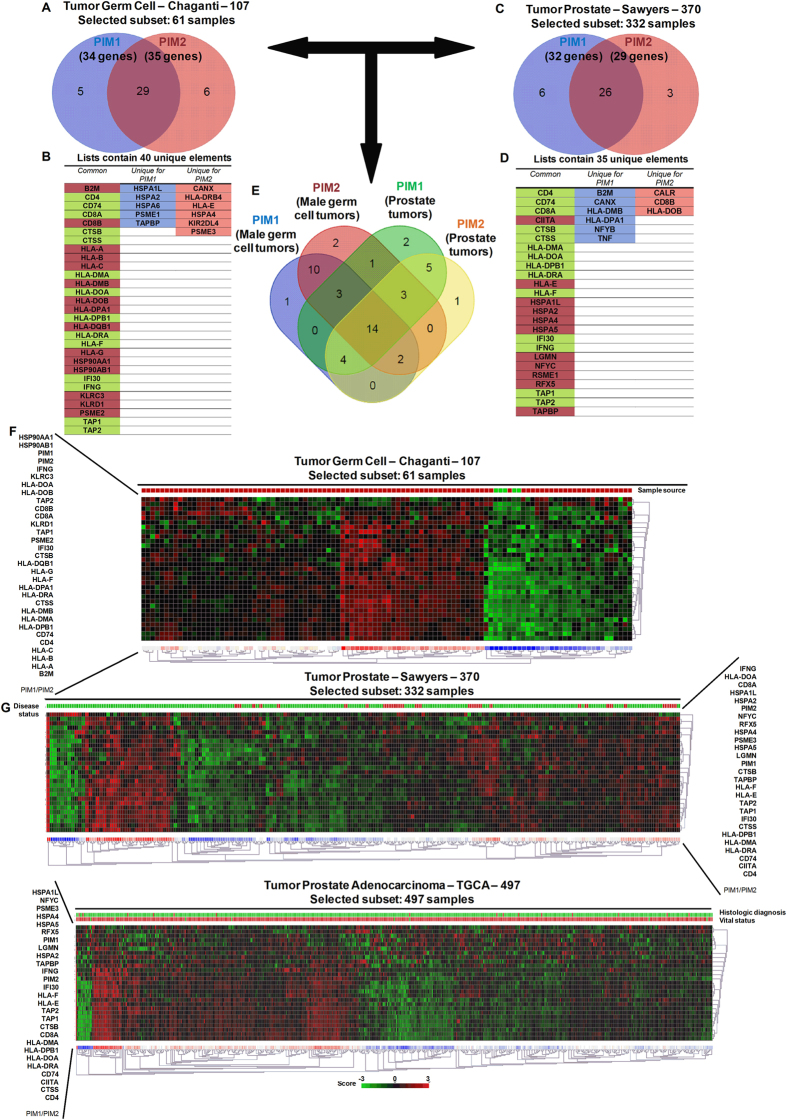
PIM overexpression correlates with an immune system-dependent genes signature in male germ cell tumors. (**A**) A Venn diagram showing the number of immune system-dependent genes that correlated with PIM1 or PIM2 overexpression in male germ cell tumors. (**B**) A list of system-dependent genes in male germ cell tumors that correlated with both PIM1 and PIM2 overexpression (common) or that was unique to either PIM1 or PIM2. (**C**) A Venn diagram showing the number of immune system-dependent genes that correlated with PIM1 or PIM2 overexpression in prostate tumors. (**D**) A list of system-dependent genes in prostate tumors that correlated with both PIM1 and PIM2 overexpression (common) or that was unique to either PIM1 or PIM2. (**E**) A Venn diagram showing the number of common immune system-dependent genes that correlated with PIM1 or PIM2 overexpression in male germ cell and prostate tumors. (**F**) The figure shows a heat map comparing immunology-related genes with PIM1 (See Supplementary Table S1) and PIM2 (See Supplementary Table S2). PIM1 and PIM2 are statistically increased in the analyzed dataset for male germ cell tumors (GSE21034) (PIM1 p = 0.06, PIM2 p = 0.05) (Control sample n = 70, Primary tumor n = 262. Unpaired t-test). We observed that approximately 45% of the patients had overexpression of either PIM1 or PIM2 in their tumors compared to the control. (**G**) The figure shows two heatmaps from different databases (GSE21034, and TCGA datasets) comparing immunology-related genes in prostate tumors with PIM1 (See Supplementary Table S3) and PIM2 (See Supplementary Table S4). Although neither PIM1 nor PIM2 are statistically increased in the analyzed dataset for prostate tumors (PIM1 p = 0.06, PIM2 p = 0.05) (Control sample n = 70, Primary tumor n = 262. Unpaired t-test), we observe that approximately 15% of the patients had overexpression of either PIM1 or PIM2 in their tumors compared to the control.

PIM1/2 kinases are weak oncogenes but undoubtedly attract immune cells to the tissue where high expression levels occur (Figs 3 and 4, and Supplementary Figures S1 and S2). To address whether a similar increase in inflammatory infiltrates is observed in human male germ cell and prostate tumors, we first analyzed the available public male germ cell tumor datasets for PIM expression and correlated these results with the inflammatory phenotype. We found a clear correlation between inflammatory markers and PIM1/2 expression (Fig. 6A, B and F, and Supplementary Tables S1 and S2). Noticeably, HLAs and membrane presentation of antigen molecules were positively correlated in human male germ cell tumors. Most of these molecules are common for both PIM1 and PIM2 (29 out of 40), although a small unique subset is found for each PIM1/2 proteins.

A similar correlation was observed in prostate tumors. Specifically, 25 out of 35 genes related to antigen presentation were common for both PIM1 and PIM2 overexpressing tumors (Fig. 6C, D and G, and Supplementary Tables S3 and S4). Interestingly, many of these genes (14 out of 25) were also common for male germ cell tumors (Fig. 6B,D and E colored in green).

These data clearly support the relevance of inflammation in tumors with PIM overexpression.

To fully confirm these conclusions, we performed qRT-PCR in PIM tg mice to test whether the inflammatory molecules were already expressed in protumor lesions (Supplementary Figures S3 and S4). We found clearly expressed HLA antigens in protumoral testis, seminal vesicles and prostate lesions in both transgenic mice, PIM1 and PIM2, (Supplementary Figure S3). Furthermore, other inflammatory antigens also present in human tumors, such as CD4, CD8a, CD74, IFNg, TAP1 or TAP2, also were activated in mice transgenic lesions (Supplementary Figure S4). Interestingly, most of these molecules were more activated in PIM1 than in PIM2 transgene, coinciding with the relative oncogenic capability found in these mice.

### PIM1 and PIM2 expression correlates with stem markers in human male germ cell and prostate tumors

It is known that inflammation may trigger dedifferentiation of tumor cells towards a more stem-like phenotype. Therefore, we tested whether PIM1 and PIM2 expression is correlated with stem markers in human male germ cell and prostate tumors. Furthermore, PIM kinases are strongly associated with chemoresistance, and it is possible that factors related to self-renewal and pluripotency such stem-like genes, which are triggered by PIM kinases, can partially explain the observed chemoresistance.

**Figure 7:**
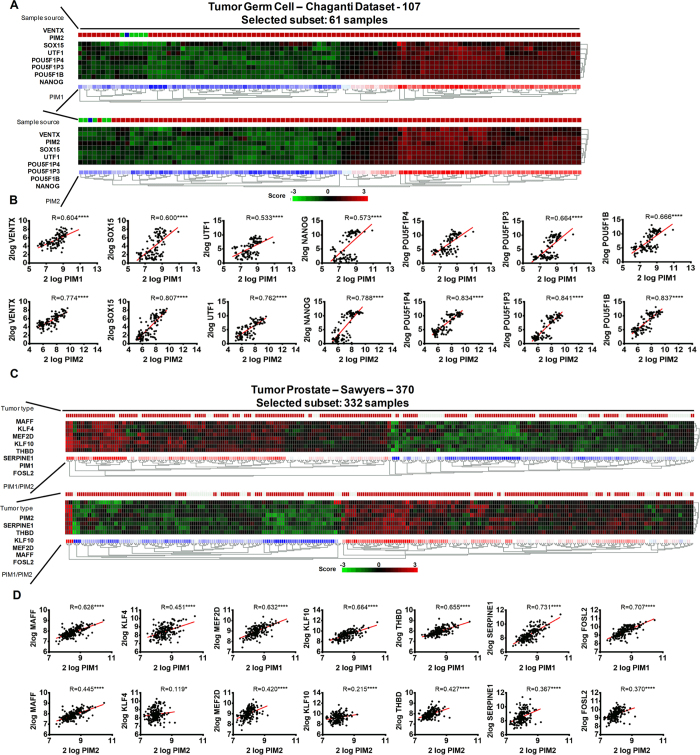
The correlation between PIM overexpression in human male germ cell and prostate tumors and the stem-dependent genes signature. (**A**) A heat map of the PIM kinases-derived signature in the adult male germ cell tumors dataset (GSE3218). This dataset shows a percentage of the patients with a clear positive PIM kinases-derived stem signature, which included more than 45% of patients (Heat-map is sorted by City-block distances). (**B**) Analysis of the correlation between PIM1/2 overexpression and factors related to self-renewal and pluripotency, such stem-like genes in germ cell tumors. To study this, we analyzed the correlation between PIM1/2 expression and stem-like gene expression and found a significant positive correlation with VENTX, SOX15, UTF1, NANOG, POU5F1P4, POU5F1P3, and POU5F1B. (**C**) The figure shows a heat map of the correlation between PIM1/2 expression in human prostate tumors and the previous stem-like gene signature. (**D**) Analysis of the correlation of PIM1/2 overexpression with factors related to self-renewal and pluripotency, such stem-like genes in prostate tumors. To this end, we analyzed the correlation between PIM1/2 expression and stem-like gene expression and found a significant positive correlation with KLF4, KLF10, FOSL2, SERPINE1, THBD, MEF2D, and MAFF.

To this end, we selected either PIM1 or PIM2 and explored the correlation between the expression of these kinases and the expression of stem-like genes activated by their expression. We found a correlation between PIM1/2 expression and VENTX, SOX15, UTF1, NANOG, POU5F1P4, POU5F1P3, and POU5F1B expression in male germ cell tumors (Fig. 7A and B). When we compared the PIM kinases-derived signature to the adult male germ cell tumors dataset (GSE3218), we observed that more than 45% of tumors are positive for the PIM kinases-derived signature (Fig. 7A).

In human prostate tumors, it was also possible to detect a small subset of tumors with a clear increase in the levels of PIM1/2 transcription. This subset of tumors with high PIM1/2 constitutes approximately 15% of tumors and is related to tumors with a stem signature (Fig. 7C and D). In this case, KLF4, KLF10, FOSL2, SERPINE1, THBD, MEF2D, and MAFF, showed a clear correlation with increased levels of PIM1/2 (Fig. 7C and D). Interestingly, while in germ cell tumors, NANOG is a relevant transcript, in prostate tumors, KLF4 is a relevant stem marker. This finding along with the different signature found in each tumor type suggests that different pathways may be involved in controlling these phenotypes in each tumor type.

These data clearly show that PIM kinases alter the physiology of tumor cells by increasing the stem cell-like properties of these cells.

## Discussion

To explore the role of PIM1 and PIM2 kinases in cancers of the male reproductive organs, we generated conditional PIM1 and PIM2 transgenic mice that overexpress PIM1 and PIM2 in male reproductive organs, which is driven by MMTV-dependent Cre, and analyzed the contribution of these kinases to neoplastic initiation and progression. We found an increase in alterations in the male sex organs, hyperplasia, and increased proliferation in the PIM1 and PIM2 transgenic mice. The overexpression of PIM proteins also correlated with increased immune invasion in these tissues. We also found that PIM1 and PIM2 are overexpressed in a subset of human male germ cell and prostate tumors and that this overexpression correlated with clear inflammatory features and stem cell markers. Therefore, our data suggest that the overexpression of PIM1/2 kinases in the male reproductive organs induce tissue organ alterations and a large inflammatory response. This local inflammation may act synergistically with PIM kinases intracellular effectors to potentiate the process of tumorigenesis. There is also a correlation with markers of cancer stem cells, which may contribute to the therapy resistance found in tumors overexpressing PIM kinases.

PIM kinases have been implicated as prognostic factors in several types of cancer^24^,^39^. Recent studies have also correlated PIM1 kinase expression with chemoresistance in prostate cancer cells, which is a common occurrence in more aggressive, hormone-refractory prostate cancers^25^,^26^. However, overexpression of PIM1 has only a weak oncogenic effect in cells, including cells of the prostate. In a similar model but with PIM expression being driven by a PSA-dependent promoter, the combination of PIM1 with hormone treatment, or in combination with reduced PTEN levels, induced more frequent mPIN lesions and lesions of a higher grade^38^.

Similar to other transgenic or knockout models^40^,^41^,^42^,^43^, our model showed that increased expression of PIM1/2 alone, driven by the viral promoter MMTV, was not sufficient to produce adenocarcinoma; however, PIM expression clearly contributed to the observed increase in hyperplasia and other precancerous lessions. This finding is consistent with reports on cell lines that showed PIM overexpression alone was not sufficient to induce malignant transformation of benign cells but did enhance the *in vitro* and *in vivo* tumorigenic capabilities of tumor cells^44^,^45^. Similarly, mice expressing PIM1 in T-cells were more susceptible to carcinogenesis-induced T-cell lymphoma^16^,^46^. It has also been reported that transgenic mice selectively expressing human PIM3 in the liver have an increased frequency and decreased latency of hepatocellular carcinoma induced by the carcinogen diethylnitrosamine^47^. Combined with our data, these studies suggest that while PIM family members are weak oncogenes, they can contribute to tumorigenesis by selectively enhancing tumorigenic capabilities related to progression process.

The overexpression of PIM proteins clearly correlated with an increase in immune cell invasion in these tissues. We also found that PIM1 and PIM2 are overexpressed in a subset of male germ cell and prostate tumors and that this overexpression correlated with clear inflammatory features and stem cell markers. Therefore, our data suggest that the overexpression of PIM1/2 kinases is a common feature of tumors of the male reproductive organs due to tissue organ alterations and a large inflammatory response, which may act synergistically during the process of tumorigenesis. It is possible that the expression of the PIM proteins acts as an immune chemoattractant, and this immune presence then triggers neoplastic alterations. Therefore, the weak effect of PIM oncogenes observed *in vitro* might be due to the absence of complementary signals that could potentially occur in the presence of inflammation.

In this context, enhanced local inflammation could be a tumor promotion mechanism led by high levels of PIM and inducing the activation of the JAK/STAT signaling pathway. STAT proteins have been linked to the control of the development of hematopoietic cells involved in inflammation, and mediating the responses of target cells to inflammatory cytokines. This has been corroborated in several PIM-dependent *in vitro* and *in vivo* models^48^,^49^. PIM1 appears to contribute to NFκB activation upon TNF-α activation^50^ through a feedback loop, while PIM1-inhibitors prevent NFκB activation and decrease iNOS production in macrophages and decrease levels of TNFα. In this regard, high PIM levels increase the recruitment of tumor/inflammation associated macrophages, MDSCs, mast cells, and neutrophils to the target tissue, which can increase cytokines, such as IL-6, IL-1 and TNFa, and chemokines, such as CCL2 and CXCL8, locally. This local increase of cytokines surrounding tumor cells might collaborate in tumorigenesis by activating the NFkB and/or STAT3 pathways^51^.

We reported here that the high levels of PIM kinases correlated in human tumors with some pluripotency related genes such as NANOG, SOX15 or KLF4 (Fig. 7). This is consistent with other works describing that PIM phosphorylates and enhances OCT4 and MYC, which also contributes to the reprogramming of tumor cells^8^,^52^. Furthermore, other PIM-dependent pathways promoting the self-renewal of stem cells have been reported^53^,^54^,^55^,^56^. PIM kinases might influence self-renewal and reprogramming through direct phosphorylation of BCRP/ABCG2, a putative stem cell marker, which may be involved in multiple drug resistance^57^. PIM kinases might regulate androgen receptor (AR) turnover by phosphorylation^58^. Therefore, PIM1-dependent phosphorylation of AR regulates gene transcription, especially STAT3, which is prevalent in aggressive prostate cancer^27^,^59^. This effect of PIM proteins on AR may influence stem cell renewal mediated by STAT3^57^.

PIM may also influence the cancer stem cell-related phenotype by the recruitment of local inflammation observed in the tumor microenvironment. Inflammatory cytokines such as IL-1, IL-6, and IL-8 may be induced by monocytes and macrophages recruited by the tumor, and may regulate cancer stem cell self-renewal in their niches. These cytokines activate the STAT3/NF-κB pathways in tumor and stromal cells generating positive feedback loops that contribute to CSC self-renewal^60^. Growth receptors and their ligands, such as HER2, EGFR, or EGF, activate NFκB, and the pro-inflammatory cytokine IL-6 activates both NFκB and STAT3^61^. Elevated levels of IL-6 have been linked to decreased survival in breast, pancreatic, gastric, prostate, and lung cancers. Moreover, chemokine receptors and their ligands are frequently expressed in malignant cells. For example, the levels of the chemokine receptor CXCR4 correlate with the ability of primary tumors to metastasize^62^. The relevance of inflammation in CSC physiology comes from the observation that non-CSCs may revert to a stem cell-like state, even in the absence of DNA mutations, which confirms that the microenvironment is able to regulate the stem-like properties of cells^63^.

Inflammation is not only essential to cancer induction by its mutagenic effects but also for the long-term survival of the tumors by influencing the microenvironment. Activation of the NFκB and STAT3 pathways remove the immune mask set by the CSC^64^. It has been proposed that the tumorigenic potential of CD14+ cells is due to their expression of several inflammatory mediators, such us IL-6, IL-8, FGF-2, and VEGF-1^65^. In this regard, there is evidence of a role for PIM1 in iPS through the IL-6/STAT3 pathway, which enhances the proliferation of mesenchymal stem cells^66^. Furthermore, in PIM1 transgenic mice, the number of Lin−/Sca+/cKit+ hematopoietic stem/progenitor cells was increased compared to normal mice^67^. Therefore, PIM kinases may serve to attract inflammatory cells and induce stem cell self-renewal, which may engage in a positive feedback loop increasing the tumorigenic potential of tumor cells that overexpress PIM kinases.

In summary, PIM kinases are overexpressed in a large variety of human tumors. Both *in vitro* and *in vivo* studies have demonstrated that PIM kinases behave as oncogenes by enhancing tumor cell proliferation and protecting from apoptosis. We fully characterized the tumor response to PIM1/2 overexpression in transgenic mouse models, which corroborated their role as weak oncogenes by inducing a hyperproliferation state in the tissue of the male reproductive system. Furthermore, we found a high inflammatory response associated with PIM expression. The analysis of human germ cell and prostate tissues showed that PIM overexpressing tumors are also associated with an increased immune response and the presence of stem cell markers. We propose that PIM kinases induce a pro-inflammatory microenvironment that cooperates with PIM kinases in the tumorigenic process by either directly or indirectly modifying the CSC pool.

## Additional Information

**How to cite this article**: Jiménez-García, M. P. *et al*. The role of PIM1/PIM2 kinases in tumors of the male reproductive system. *Sci. Rep.* **6**, 38079; doi: 10.1038/srep38079 (2016).

**Publisher's note:** Springer Nature remains neutral with regard to jurisdictional claims in published maps and institutional affiliations.

## Supplementary Material

Supplementary Information
