# Supplementary material for: The role of PIM1/PIM2 kinases in tumors of the male reproductive system
Source: Sci Rep. 2016 Nov 30;6:38079. doi: 10.1038/srep38079 (PMC5128923; doi:10.1038/srep38079)

Supplementary Data

THE ROLE OF PIM1/PIM2 KINASES IN TUMORS OF THE MALE REPRODUCTIVE SYSTEM

Manuel Pedro Jiménez-García, Antonio Lucena-Cacace, María José Robles-Frías, Maja Narlik-Grassow, Carmen Blanco-Aparicio, Amancio Carnero

Table S1: The correlation between the expression of antigen processing and presentation (HLA and inflammation) genes and PIM1 expression in human male germ cell tumors. Database: ***GSE3218*****.**

	r-value	*P-Value*
**HLA-F**	0.562	< 0.01
**IFI30**	0.557	< 0.01
**HLA-A**	0.552	< 0.01
**CD74**	0.531	< 0.01
**HLA-A**	0.530	< 0.01
**IFNG**	0.488	< 0.01
**HLA-DPA1**	0.477	< 0.01
**CD4**	0.473	< 0.01
**CTSS**	0.465	< 0.01
**HLA-C**	0.458	< 0.01
**HLA-G**	0.458	< 0.01
**HLA-DPB1**	0.451	< 0.01
**CTSB**	0.449	< 0.01
**HLA-DQB1**	0.441	< 0.01
**HLA-B**	0.441	< 0.01
**TAP1**	0.435	< 0.01
**B2M**	0.430	< 0.01
**HLA-DRA**	0.425	< 0.01
**HLA-DMA**	0.420	< 0.01
**KLRD1**	0.417	< 0.01
**PSME2**	0.401	< 0.01
**CD8B**	0.400	< 0.01
**TAP2**	0.389	< 0.01

**CD8A**	0.365	< 0.01
**KLRC3**	0.351	< 0.01
**HLA-DMB**	0.347	< 0.01
**TAPBP**	0.334	< 0.01
**HSP90AB1**	0.321	< 0.01
**HSPA6**	0.307	< 0.01
**HLA-DOB**	0.300	< 0.01
**PSME1**	0.297	< 0.01
**HLA-DOA**	0.273	< 0.01
**HSP90AA1**	0.267	< 0.01
**HSPA2**	-0.239	< 0.05
**HSPA1L**	-0.221	< 0.05

Table S2: The correlation between the expression of antigen processing and presentation (HLA and inflammation) genes and PIM2 expression in human male germ cell tumors. Database: *GSE3218*.

	r-value	*P-Value*
**IFNG**	0.598	< 0.01
**HLA-F**	0.496	< 0.01
**IFI30**	0.463	< 0.01
**HLA-E**	0.445	< 0.01
**CD74**	0.441	< 0.01
**HSPA4**	0.437	< 0.01
**TAP1**	0.408	< 0.01
**PSME2**	0.406	< 0.01
**HSP90AB1**	0.404	< 0.01
**HLA-G**	0.383	< 0.01
**CD8B**	0.380	< 0.01
**KLRC3**	0.377	< 0.01
**HLA-DPA1**	0.375	< 0.01
**HSP90AA1**	0.367	< 0.01
**KLRD1**	0.366	< 0.01
**CTSS**	0.363	< 0.01
**HLA-A**	0.363	< 0.01
**HLA-B**	0.358	< 0.01
**HLA-DRA**	0.356	< 0.01
**CD4**	0.351	< 0.01
**PSME3**	0.347	< 0.01
**HLA-C**	0.345	< 0.01
**CD8A**	0.309	< 0.01
**HLA-DQB1**	0.304	< 0.01
**HLA-DPB1**	0.304	< 0.01
**CTSB**	0.299	< 0.01
**HLA-DMB**	0.298	< 0.01

**HLA-DMA**	0.287	< 0.01
**B2M**	0.222	< 0.01
**TAP2**	0.217	< 0.01
**HLA-DRB4**	0.197	< 0.01
**HLA-DOA**	0.196	< 0.01
**CANX**	-0.194	< 0.05

Table S3: The correlation between the expression of antigen processing and presentation (HLA and inflammation) genes and PIM1 expression in human prostate tumors. Database: Sawyer gse21034geot370.

	r-value	*P-Value*
**HLA-DMA**	0.555	2.7e-31
**TAPBP**	0.544	7.1e-30
**CD4**	0.543	1.0e-29
**TAP2**	0.539	2.7e-29
**HLA-E**	0.537	5.6e-29
**RFX5**	0.528	6.5e-28
**TAP1**	0.524	1.6e-27
**HLA-DPB1**	0.505	2.4e-25
**CTSS**	0.504	3.0e-25
**IFI30**	0.477	1.8e-22
**NFYC**	0.468	1.6e-21
**HLA-DRA**	0.463	4.6e-21
**PSME3**	0.459	1.2e-20
**HLA-F**	0.451	6.0e-20
**CD74**	0.450	6.9e-20
**HSPA4**	0.433	2.4e-18
**LGMN**	0.420	3.0e-17
**CIITA**	0.417	5.7e-17
**HLA-DPA1**	0.407	3.3e-16
**PSME1**	0.407	3.4e-16
**CTSB**	0.377	6.0e-14
**HLA-DOA**	0.363	5.4e-13
**HLA-DMB**	0.359	1.1e-12
**CANX**	0.350	4.4e-12
**HSPA2**	0.322	2.3e-10
**NFYB**	0.314	6.5e-10
**HSPA1L**	0.299	4.2e-09

**HSPA5**	0.290	1.3e-08
**B2M**	0.286	2.0e-08
**CD8A**	0.280	4.2e-08
**TNF**	-0.166	1.4e-03
**IFNG**	-0.165	1.5e-03

Table S4: The correlation between the expression of antigen processing and presentation (HLA and inflammation) genes and PIM2 expression in human prostate tumors. Database: Sawyer gse21034geot370.

	r-value	*P-Value*
**TAP2**	0.545	4.9e-30
**PSME1**	0.524	1.6e-27
**CIITA**	0.521	3.5e-27
**TAPBP**	0.515	2.0e-26
**HSPA2**	0.492	5.3e-24
**HLA-DOA**	0.481	7.2e-23
**CD4**	0.471	8.3e-22
**RFX5**	0.453	3.8e-20
**CD8A**	0.437	1.2e-18
**HLA-F**	0.424	1.3e-17
**TAP1**	0.420	2.9e-17
**HLA-E**	0.417	5.3e-17
**HLA-DMA**	0.403	7.0e-16
**PSME3**	0.392	5.1e-15
**IFI30**	0.382	2.6e-14
**HSPA1L**	0.323	2.0e-10
**NFYC**	0.313	7.0e-10
**LGMN**	0.303	2.7e-09
**CTSB**	0.283	3.0e-08
**CALR**	0.252	9.3e-07
**CTSS**	0.239	3.3e-06
**HSPA4**	0.239	3.4e-06
**CD74**	0.221	1.7e-05
**HLA-DPB1**	0.190	2.4e-04
**HLA-DRA**	0.189	2.5e-04
**HLA-DOB**	0.172	8.9e-04
**CD8B**	0.161	1.9e-03

**HSPA5**	0.156	2.7e-03
**IFNG**	-0.166	1.3e-03

Supplemental figure 1. Immunohistological characterization of immune infiltration in the testes from MMTV-Cre/PIM1 and MMTV-Cre/PIM2 transgenic models. Pathological analysis of the testes was performed by using immune mouse markers in order to determine whether the infiltration is lymphoid (CD3 and CD45) or macrophage (F4/80) origin. Scale bars indicate 50 μm (40X) and all of the images were captured using the Microscope Olympus BX-61.

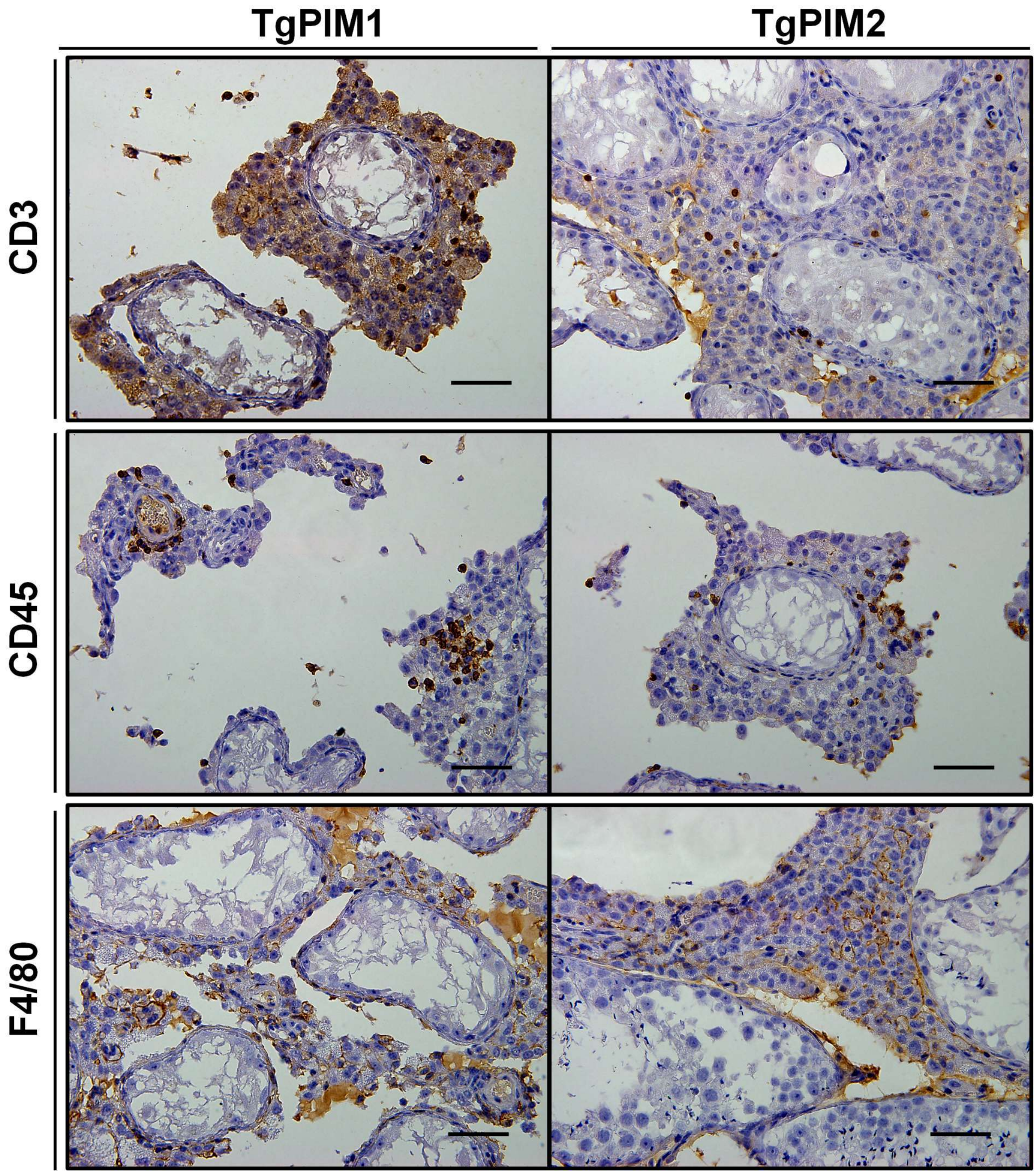

Supplemental figure 2. Immunohistological characterization of immune infiltration in the seminal vesicle and prostate from MMTV-Cre/PIM1 and MMTV-Cre/PIM2 transgenic models. The figure shows representative images of immune infiltration (marked with arrows) in different male reproductive organs (seminal vesible and prostate) in transgenic mice. Immuno-pathological analysis of the seminal vesicle and prostate was performed by using both proliferation markers (Ki67) and immune mouse markers in order to determine whether the infiltration is lymphoid (CD3 and CD45) or macrophage (F4/80) origin. The figure shows representative images of immune infiltration (marked with arrows in H&E), and macrophage cells (marked with arrows in F4/80). Scale bars of H&E indicate 200 µm (10X) and the rest of markers indicate 50 µm (40X). All of the images were captured using the Microscope Olympus BX-61.

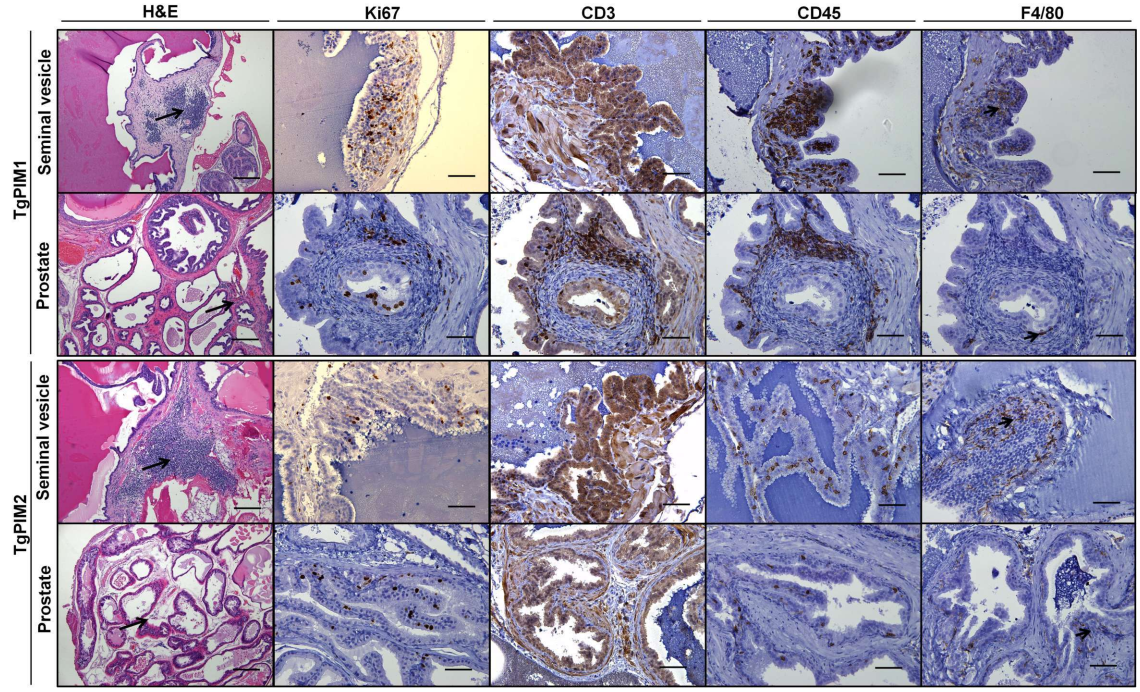

Supplemental figure 3. Pro-inflammatory Major Histocompatibility characterization by qPCR. The figure shows a number of mouse Major Histocompatibility Complex genes (MHC, also called H2 in mouse) that were correlated with PIM overexpression in human male germ cell and prostate tumors in figure 6. We focused on the ones with mouse orthologs: HLA-DMA (H2.DMA), HLA-DRA (H2.A), HLA-DPB1 (CD74, measured in supplemental figure 4), and also H2.K and H2.E, from classical MHC class I and II, respectively. They were measured by qRT-PCR in testicle **(A)**, seminal vesicle **(B)** and prostate **(C)**, comparing TgPIM1 and TgPIM2 mice to WT mice. The p-value was obtained using a one-tailed student's t-test. (*p < 0.05), (**p < 0.01), and (***p < 0.001).

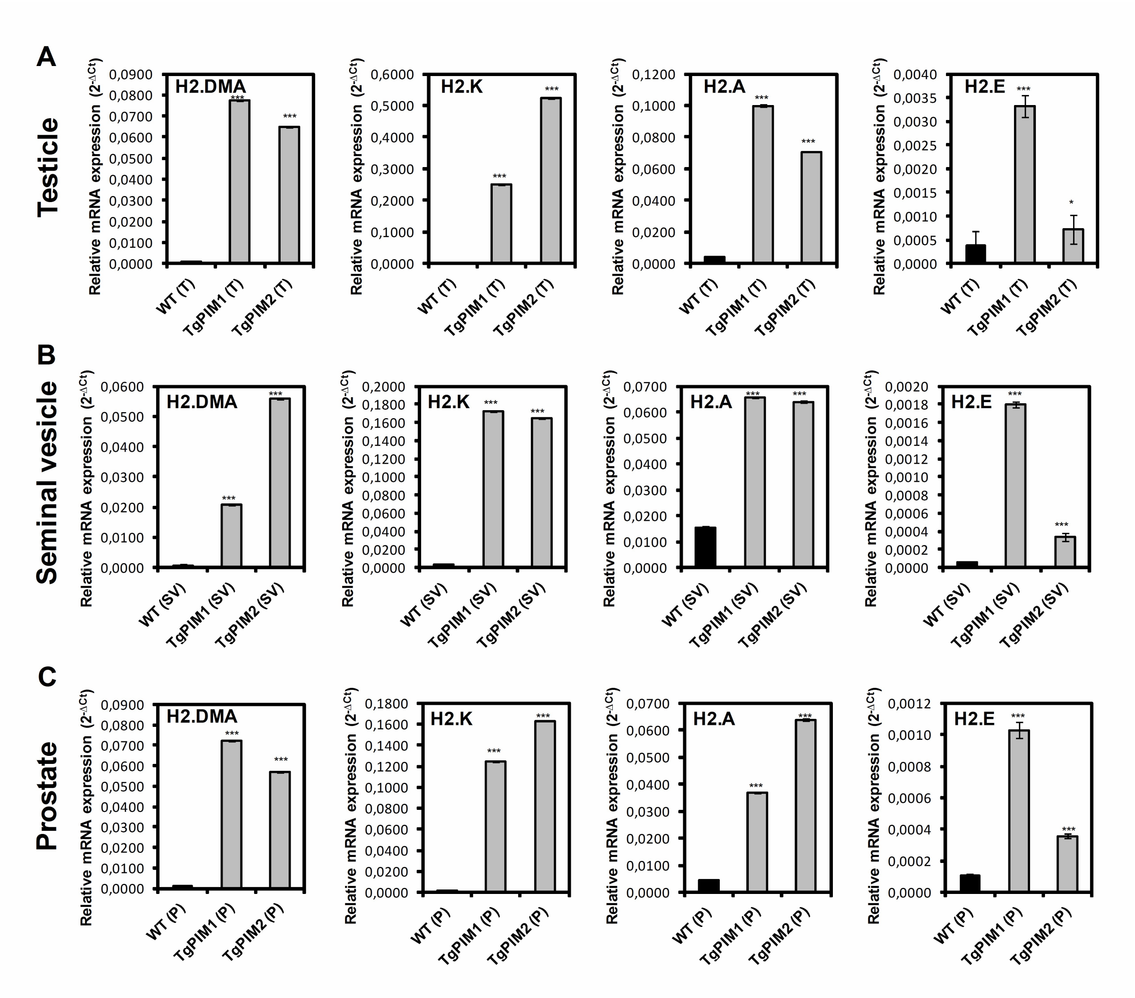

Supplemental figure S4. Pro-inflammatory microenvironment PIM-dependent characterization by q-RT-PCR. The figure shows a number of immune system-dependent genes expression in mouse that were correlated with PIM overexpression in human male germ cell and prostate tumors (CD4, CD8a, CD74, Ifng, Tap1 and Tap2) which it are reported in figure 6. They were measured by qRT-PCR in testicle **(A)**, seminal vesicle **(B)** and prostate **(C)**, comparing TgPIM1 and TgPIM2 mice to WT mice. The p-value was obtained using a one-tailed student's t-test. (*p < 0.05), (**p < 0.01), and (***p < 0.001).

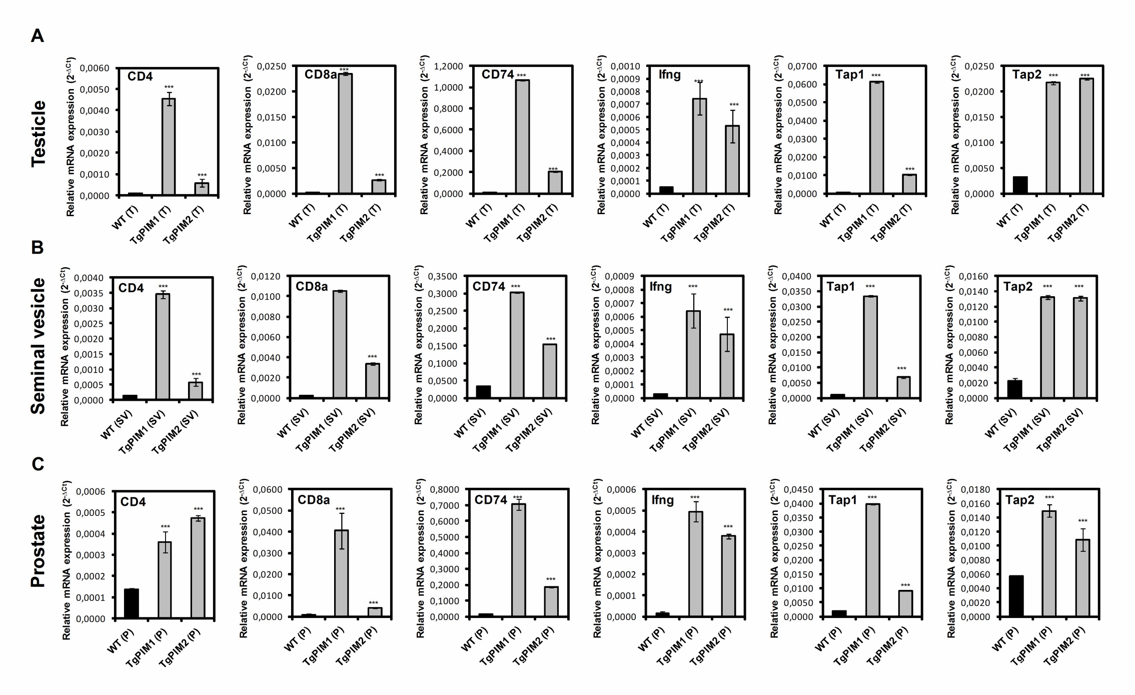

Supplement: Supplementary Information [file srep38079-s1.pdf]
